# Supplementary material for: Pregnancy Management in HIV Viral Controllers: Twenty Years of Experience
Source: Pathogens. 2024 Apr 10;13(4):308. doi: 10.3390/pathogens13040308 (PMC11054990; doi:10.3390/pathogens13040308)
Supplement: Supplementary file 1 [file pathogens-13-00308-s001.zip › Suplementary Table S1.pdf]

Supplementary table S1: Summary of HIV-1 RNA quantification assays used in reported cases

**Assay**	**Manufacturer**	**Lower limit of quantification (LLoQ)**
**RealTime**	Abbott	40
**Bayer's VERSANT bDNA 3.0**	Bayer	75
**Quantiplex 3.0**	Chiron	50
**Amplicor HIV-1 Monitor 1.5 sensitive**	Roche	400
**Amplicor HIV-1 Monitor 1.5 ultra-sensitive**	Roche	50
**Ampliprep/COBAS Taqman 1**	Roche	40
**Ampliprep/COBAS Taqman 2**	Roche	20
**Aptima HIV 1 Quant Dx**	Hologic	30
