# Supplementary material for: Pregnancy Management in HIV Viral Controllers: Twenty Years of Experience
Source: Pathogens. 2024 Apr 10;13(4):308. doi: 10.3390/pathogens13040308 (PMC11054990; doi:10.3390/pathogens13040308)
Supplement: Supplementary file 1 [file pathogens-13-00308-s001.zip › Suplementary Table S2.pdf]

**Summary Table S2** | Summary table of baseline characteristics, immune parameters, ART regime, delivery and postpartum infant HIV status

| | | | | | | Co-morbidities | | | | | | | | Immune Parameters | | | | | | | | | | | | | | | | | | | |
|---|---|---|---|---|---|---|---|---|---|---|---|---|---|---|---|---|---|---|---|---|---|---|---|---|---|---|---|---|---|---|---|---|---|
| ***Patient (n =28)*** | **Pregnancy Number** | **Year of Delivery** | **Age at booking /yrs** | **Diagnosed in current pregnancy** | **Region** | Hep B | Hep C | Latent TB | Hypertensive Disorder | Type 2 DM | Mental Health Diagnosis | **Co-medication** | **Para** | CD4+ at diagnosis /mcL | CD4+ in this pregnancy /mcL | CD8+ in this pregnancy /mcL | CD4+: CD8+ ratio | HIV VL repeated on alternative assay | Maternal proviral DNA result available | Maternal DNA amplified | Detectable VL in pregnancy | VL during pregnancy copies/ mL | **ART in pregnancy** | **Gestation started ART /weeks** | **GA at delivery /weeks** | **Mode of delivery** | **IV ZDV at birth** | **VL at delivery copies/ mL** | **Infant received 4 weeks ZDV** | **ART stopped PP** | **Mode of Feeding** | **12-week DNA or RNA available** | **Infant Ab status available** |
| ***1*** | 1 | 1999 | 18 | No | United Kingdom | No | No | No | No | No | No | No | Nulliparous | 570 | 670 | 260 | 1.9 | Yes | No | - | Yes | <50; <500; 579; <100; <50 | ZDV | 30.0 | 39.0 | PLCS | Yes | <50 | Yes | Yes | Formula fed | Yes, negative | Yes, negative |
| | 2 | 2001 | 19 | No | | No | No | No | No | No | No | No | Multiparous | | 740 | 460 | 1.7 | - | No | - | No | <50 | ZDV | 32.0 | 38.0 | PLCS | Yes | <50 | Yes | Yes | Formula fed | Yes, negative | Yes, negative |
| | 3 | 2017 | 36 | No | United Kingdom | No | No | No | No | No | Yes | Yes | Multiparous | | 594 | 434 | 1.4 | - | No | - | No | <20; <20; <20; <20; <20; <20; <20 | MRV/FTC/TDF | 0.0 | 38.0 | PLCS | N/A | <20 | Yes | No | Breast fed | Yes, negative | Yes, negative |
| | 4 | 2021 | 40 | No | | No | No | No | No | No | Yes | Yes | Multiparous | | 726 | 459 | 1.6 | - | No | - | No | <20; <20; <20; <20; <20; <20; <20 | MRV/FTC/TDF | 0.0 | 38.0 | PLCS | N/A | <20 | Yes | No | Formula fed | Yes, negative | Yes, negative |
| ***2*** | 1 | 1999 | 32 | Yes | Ethiopia | No | No | No | No | No | No | No | Nulliparous | 500 | 500 | 330 | 1.5 | Yes | Yes | not detected | - | - | ZDV | 28.0 | 38.0 | PLCS | - | - | Yes | Yes | - | - | Yes, negative |
| | 2 | 2004 | 37 | No | | No | No | No | No | No | No | No | Multiparous | | 500 | 330 | 1.5 | - | Yes | not detected | No | <50; <50; <50 | ZDV | 28.0 | 38.0 | PLCS | Yes | <50 | Yes | Yes | Formula fed | Yes, negative | Yes, negative |
| ***3*** | 1 | 2000 | 30 | Yes | - | No | No | No | No | No | No | No | Nulliparous | 350 | 640 | 520 | 1.2 | Yes | No | | Yes | <50; <40; 179; <50 | ZDV | 32.0 | 38.0 | PLCS | N/A | <50 | Yes | Yes | Formula fed | Yes, negative | Yes, negative |
| | 2 | 2003 | 33 | No | | No | No | No | No | No | No | No | Multiparous | | 660 | 660 | 1 | - | No | - | Yes | <50; 51; <50; <50; <50; <50; <50 | ZDV | 28.0 | 37.0 | PLCS | N/A | <50 | Yes | Yes | Formula fed | Yes, negative | Yes, negative |
| ***4*** | 1 | 2000 | 25 | Yes | South Africa | No | No | No | Yes | No | No | No | Nulliparous | 460 | 770 | 1170 | 0.5 | Yes | Yes | detected | No | <50; <50; <50 | ZDV | 32.0 | 34.0 | emLCS | Yes | <50 | Yes | Yes | Formula fed | Yes, negative | Yes, negative |
| | 2 | 2006 | 32 | No | | No | No | No | Yes | No | No | No | Multiparous | | 570 | 960 | 0.5 | - | Yes | detected | Yes | 289; 237; 223; 145; 89; 78; 54 | ZDV | 24.0 | 38.0 | PLCS | N/A | 54 | Yes | Yes | Formula fed | Yes, negative | Yes, negative |
| ***5*** | 1 | 2001 | - | Yes | Sierra Leone | No | No | No | No | Yes | No | Yes | Nulliparous | - | - | - | - | - | Yes | ,not detected | - | - | ZDV | 28.0 | 38.0 | PLCS | - | - | Yes | Yes | Formula fed | - | Yes, negative |
| | 2 | 2005 | - | No | | No | No | No | No | Yes | No | Yes | Multiparous | - | - | - | - | - | Yes | not detected | - | - | ZDV | 28.0 | 38.0 | PLCS | - | - | Yes | Yes | Formula fed | - | Yes, negative |
| | 3 | 2011 | - | No | | No | No | No | No | Yes | No | Yes | Multiparous | - | 682 | 342 | 2 | - | Yes | not detected | - | - | ZDV | 22.6 | 38.0 | PLCS | Yes | - | Yes | Yes | Formula fed | Yes, negative | Yes, negative |
| | 4 | 2019 | - | No | | No | No | No | No | Yes | No | Yes | Multiparous | 497 | 780 | 371 | 2.1 | Yes | Yes | not detected | Yes | 105; 44; <20; 0; 0; <20 | ZDV | 20.0 | 38.0 | PLCS | No | <20 | No | Yes | Formula fed | Yes, negative | No |

| | | | | | | | | | | | | | | | | | | | | | | | | | | | | | | | | | |
|---|---|---|---|---|---|---|---|---|---|---|---|---|---|---|---|---|---|---|---|---|---|---|---|---|---|---|---|---|---|---|---|---|---|
| **6** | 1 | 2001 | 31 | Yes | Ethiopia | No | No | No | No | No | No | No | Nulliparous | 486 | 519 | - | - | Yes | No | - | No | <50 | ZDV | 32.0 | 36.0 | PLCS | N/A | <50 | - | Yes | Formula fed | - | - |
| **7** | 1 | 2002 | 31 | No | Zimbabwe | No | No | No | No | No | No | No | Nulliparous | 852 | 852 | - | - | Yes | Yes | not detected | No | <50; <50 | ZDV | 26.0 | 40.0 | PLCS | Yes | <50 | Yes | Yes | Formula fed | Yes, negative | Yes, negative |
| | 2 | 2006 | 34 | No | | No | No | No | No | No | No | No | Multiparous | | 1227 | - | - | - | Yes | not detected | No | <50; <50; <50; <50 | ZDV | 30.0 | 38.0 | PLCS | Yes | - | Yes | Yes | Formula fed | Yes, negative | Yes, negative |
| **8** | 1 | 2003 | 25 | No | Macedonia | No | No | No | No | No | No | No | Nulliparous | 457 | 968 | - | - | Yes | No | - | No | <50; <50; <50; <50; <50 | ZDV | 32.0 | 39.0 | PLCS | No | <50 | Yes | Yes | Formula fed | - | Yes, negative |
| | 2 | 2005 | 27 | No | | No | No | No | No | No | No | No | Nulliparous | | 1159 | - | - | - | No | - | No | <50; <50; <50; <50; <50 | ZDV | 34.0 | 38.0 | PLCS | No | <50 | Yes | Yes | Formula fed | - | Yes, negative |
| **9** | 1 | 2004 | 33 | Yes | Tanzania | No | No | No | No | No | No | No | Nulliparous | 360 | 306 | - | - | Yes | No | - | No | <50; <50; <50 | ZDV /FTC/NFV | 28.0 | 38.0 | PLCS | Yes | <50 | Yes | Yes | Formula fed | Yes, negative | Yes, negative |
| **10** | 1 | 2005 | 33 | No | United Kingdom | No | No | No | No | No | No | No | Nulliparous | 688 | 901 | - | - | Yes | Yes | detected | No | <50; <50; <50; <50; <50 | ZDV | 24.0 | 38.0 | PLCS | No | <50 | Yes | Yes | Formula fed | Yes, negative | Yes, negative |
| | 2 | 2006 | 34 | No | | No | No | No | No | No | No | No | Multiparous | | 979 | - | - | - | Yes | detected | No | <50; <50; <50; <50; <50 | ZDV | 24.0 | 39.0 | PLCS | No | <50 | Yes | Yes | - | - | Yes, negative |
| **11** | 1 | 2005 | 26 | Yes | Ivory Coast | No | No | No | No | No | No | No | Nulliparous | 551 | 685 | - | - | Yes | Yes | detected | Yes | <50; 205 | ZDV /3TC/KAL | 31.0 | 39.0 | SVD | No | 205 | Yes | - | Formula fed | Yes, negative | Yes, negative |
| **12** | 1 | 2005 | 26 | No | Nigeria | No | No | No | No | No | No | No | Nulliparous | 708 | 670 | - | - | Yes | No | - | No | <50; <50; <50; <50; <50; <50 | ZDV | 28.0 | 40.0 | PLCS | No | <50 | Yes | Yes | Formula fed | Yes, negative | Yes, negative |
| **13** | 1 | 2006 | 26 | No | United Kingdom | No | Yes | No | No | No | No | No | Nulliparous | 752 | 752 | - | - | Yes | Yes | detected | No | <50 <40 | ZDV | 24.0 | 38.0 | SVD | No | <40 | Yes | Yes | Formula fed | Yes, negative | No |
| | 2 | 2008 | 28 | No | | No | Yes | No | No | No | No | No | Multiparous | | 1023 | - | - | - | Yes | detected | Yes | 87; <40; <40; <40 | ZDV | 24.0 | 38.0 | PLCS | No | <40 | Yes | Yes | Formula fed | Yes, negative | No |
| | 3 | 2012 | - | No | | No | Yes | No | No | No | No | No | Multiparous | | 1121 | 968 | 1.2 | Yes | Yes | detected | No | 29; <20 | ZDV | 20.4 | 38.0 | PLCS | No | <20 | Yes | Yes | Formula fed | Yes, negative | No |
| **14** | 1 | 2006 | 31 | Yes | Nigeria | No | No | No | No | No | No | No | Nulliparous | 638 | 1119 | - | - | Yes | No | - | Yes | 95; <50; <50; <50 | TDF/3TC/KAL | 24.0 | 39.0 | SVD | No | <50 | Yes | Yes | Formula fed | Yes, negative | Yes, negative |
| **15** | 1 | 2006 | 27 | Yes | South Africa | No | No | No | No | No | No | No | Nulliparous | 826 | 947 | - | 1.5 | Yes | Yes | detected | No | <50; <50; <50; <50 | ABC/3TC/ZDV | 32.0 | 41.0 | SVD | No | <50 | Yes | Yes | Formula fed | Yes, negative | Yes, negative |
| **16** | 1 | 2007 | 22 | Yes | Bangladesh | No | No | No | No | No | No | No | Nulliparous | 741 | 784 | - | 1.3 | Yes | Yes | detected | No | 0; 0; 0; 0 | ZDV | 29.0 | 41.0 | SVD | No | 0 | Yes | Yes | Formula fed | Yes, negative | Yes, negative |

| | | | | | | | | | | | | | | | | | | | | | | | | | | | | | | | | | |
|---|---|---|---|---|---|---|---|---|---|---|---|---|---|---|---|---|---|---|---|---|---|---|---|---|---|---|---|---|---|---|---|---|---|
| | 2 | 2011 | 26 | No | | No | No | No | No | No | No | No | Multiparous | | 784 | - | - | - | Yes | not detected | Yes | 80; <40; 0; 211; 55; 0 | ZDV | 30.0 | 40.0 | emLCS | No | 0 | Yes | Yes | Formula fed | Yes, negative | Yes, negative |
| 17 | 1 | 2007 | 27 | Yes | Poland | No | No | No | No | No | No | No | Nulliparous | 778 | 697 | - | - | Yes | -No | - | Yes | 0; <50; <50; 6084; <40 <50 | ZDV | 28.0 | 32.0 | emLCS | Yes | <50 | Yes | Yes | Formula fed | Yes, negative | Yes, negative |
| 18 | 1 | 2007 | 33 | Yes | Nigeria | Yes | No | No | No | No | No | No | Nulliparous | 357 | 335 | - | - | - | Yes | detected | No | <40; <40 | TDF/3TC/KAL | 33.0 | 41.0 | SVD | No | <40 | Yes | Yes | - | Yes, negative | Yes, negative |
| 19 | 1 | 2008 | 30 | Yes | Nigeria | No | No | No | No | No | No | No | Nulliparous | 504 | 547 | - | - | Yes | Yes | not detected | Yes | 100; <50; <50 | ZDV | 28.0 | 39.0 | PLCS | No | <50 | - | No | Formula fed | Yes, negative | Yes, negative |
| 20 | 1 | 2008 | 35 | No | Eritrea | No | No | No | No | No | No | No | Nulliparous | 430 | 530 | 700 | 0.7 | Yes | Yes | detected | No | <50; <50 | ZDV | 28.0 | 39/0 | emLCS | Yes | <50 | Yes | Yes | - | Yes, negative | Yes, negative |
| 21 | 1 | 2008 | 32 | No | Kenya | No | No | No | No | No | No | Yes | Nulliparous | 977 | 502 | - | - | Yes | Yes | not detected | Yes | <40; 173; 88; <50; 0; <40 | ZDV | 28.0 | 38.0 | PLCS | Yes | <40 | Yes | Yes | Formula fed | Yes, negative | Yes, negative |
| 22 | 1 | 2009 | 34 | Yes | Rwanda | No | No | No | No | No | No | No | Nulliparous | 931 | 1043 | - | - | Yes | Yes | not detected | No | <40; <40; <40; <40 | ZDV | 26.0 | 39.0 | SVD | No | <40 | Yes | Yes | - | Yes, negative | - |
| 23 | 1 | 2011 | 30 | Yes | Nigeria | No | No | Yes | No | No | No | Yes | Nulliparous | 776 | 743 | 434 | 1.7 | Yes | Yes | not detected | No | <40; <40; <40; <40; <40; <40 | ABC/3TC/ZDV | 24.0 | 41.0 | PLCS | No | <40 | Yes | Yes | Formula fed | Yes, negative | No |
| | 2 | 2013 | 32 | No | | No | No | No | No | No | No | No | Multiparous | | 612 | 287 | - | - | Yes | not detected | No | 0; 0; 0; 0; 0; 0 | ABC/3TC/ZDV | 25.0 | 38.0 | PLCS | No | 0 | Yes | Yes | Formula fed | Yes, negative | Yes, negative |
| 24 | 1 | 2011 | 33 | No | South Africa | No | No | No | No | No | No | No | Nulliparous | 770 | 768 | - | - | Yes | Yes | not detected | No | 0; 0; <40; 0 | ABC/3TC/ZDV | 28.0 | 41.0 | PLCS | No | 0 | Yes | Yes | Formula fed | Yes, negative | - |
| 25 | 1 | 2012 | 33 | No | Nigeria | No | No | No | No | No | No | No | Nulliparous | 514 | 514 | 582 | 0.9 | Yes | Yes | detected | No | <40; 0; 0; 0 | TDF/FTC/ATV/r | 13.0 | 40.0 | emLCS | No | 0 | Yes | Yes | Formula fed | Yes, negative | Yes, negative |
| | 2 | 2014 | 35 | No | | No | No | No | No | No | No | No | Multiparous | | 393 | - | - | Yes | Yes | detected | No | 0; 0; 0; 0; 0 | ATV/r/FTC/TDF | 29.4 | 40.0 | VBAC | No | 0 | Yes | Yes | Formula fed | Yes, negative | Yes, negative |
| | 3 | 2018 | 39 | No | | No | No | No | No | No | No | No | Multiparous | | 412 | 607 | 0.7 | - | Yes | detected | No | 0; 0; 0; 0; 0; 0 | RAL/FTC/TDF | 7.4 | 38.0 | emLCS | No | 0 | Yes | Yes | Formula fed | Yes, negative | Yes, negative |
| 26 | 1 | 2012 | - | No | Jamaica | No | No | No | No | No | No | No | Nulliparous | 651 | 972 | 699 | 1.4 | Yes | Yes | detected | Yes | 52; 38; <20; <20 | ZDV | 23.0 | 40.0 | SVD | No | <20 | Yes | Yes | Formula fed | Yes, negative | Yes, negative |
| 27 | 1 | 2016 | - | Yes | Nigeria | Yes | No | No | No | No | No | No | Nulliparous | 455 | 455 | 1120 | 0.4 | Yes | Yes | detected | No | <20; <20; <20; <20 | RPV/FTC/TDF | 18.3 | 37.0 | emLCS | No | <20 | Yes | No | Formula fed | Yes, negative | Yes, negative |
| 28 | 1 | 2019 | - | No | Zambia | No | No | No | Yes | No | No | Yes | Nulliparous | 952 | 975 | 875 | 1.1 | Yes | No | - | Yes | 78; 41; <20; <20 | RPV/TDF/FTC | 22.7 | 39.0 | SVD | No | <20 | Yes | No | Mixed | Yes, negative | - |

| | | | | | | | | | | | | | | | | | | | | | | | | | | | | | | | | | |
|---|---|---|---|---|---|---|---|---|---|---|---|---|---|---|---|---|---|---|---|---|---|---|---|---|---|---|---|---|---|---|---|---|---|
| ***29*** | 1 | 2012 | 24 | Yes | Angola | No | No | No | No | No | No | Yes | Nulliparous | 1 | 2012 | - | - | - | - | Yes | No | <50 | ATV/r/ZDV/3TC | - | 39.0 | PLCS | - | <50 | - | Yes | Formula fed | Yes, negative | - |
| | 2 | 2014 | 27 | | | No | No | No | No | No | No | Yes | Multiparous | 2 | 2014 | - | 1050 | - | - | - | No | <50; <50; <50; <50 | ZDV | 28.0 | | VBAC | No | <50 | Yes | Yes | Formula fed | Yes, negative | Yes, negative |
| | 3 | 2021 | 33 | | | No | No | No | No | No | No | Yes | Multiparous | 3 | 2021 | - | 1030 | - | - | - | No | <50; <50; <50; <50 | RAL/FTC/TDF | | | VBAC | No | <50 | 14 days | Yes | Formula fed | Yes, negative | Yes, negative |

Abbreviations: HIV, Human Immunodeficiency Virus-1; Hep B, hepatitis B; Hep C, hepatitis C; TB, Tuberculosis; CD4+, cluster of differentiation; CD8+, cluster of differentiation 8; VL, viral load; ART, antiretroviral therapy; GA, gestational age; IV, intravenous; ZDV, Zidovudine; N/A, not applicable; PP, postpartum; Ab, antibody; MRV, Maraviroc; FTC, Emtricitabine; TDF, Tenofovir disoproxil fumarate; 3TC, Lamivudine; KAL, kaletra (lopinavir/ritonavir); ABC, Abacavir; ATV/r, Atazanavir/ritonavir; RAL, Raltegravir; RPV, Rilpivirine; PLCS, planned caesarean section; emLCS, emergency caesarean section; SVD, spontaneous vaginal delivery.
